# Supplementary material for: Association of Gene Variants for Mechanical and Metabolic Muscle Quality with Cardiorespiratory and Muscular Variables Related to Performance in Skiing Athletes
Source: Genes (Basel). 2022 Oct 5;13(10):1798. doi: 10.3390/genes13101798 (PMC9602077; doi:10.3390/genes13101798)
Supplement: Supplementary file 1 [file genes-13-01798-s001.zip › supplementarytables.pdf]

Table S1. *Genotype associations of strength.* Effect sizes and p-values of the ANOVA for factors genotype (maximally 2) x sex x gender.

main effects (>0.10)		peak torque	peak torque scaled	peak power	peak power scales
ACE_ID	effect size	0.090	0.130	0.250	0.300
	p-value	<0.001	<0.001	<0.001	<0.001
ACE_ID * Gender	effect size	<0.001	0.090	0.160	0.160
	p-value	0.660	<0.001	<0.001	<0.001
ACTN3	effect size	0.150	0.090	0.550	0.550
	p-value	<0.001	<0.001	<0.001	<0.001
ACTN3 * Gender	effect size	0.050	0.120	0.350	0.430
	p-value	0.010	<0.001	<0.001	<0.001
ACE_ID * ACTN3	effect size	0.040	0.060	0.110	0.190
	p-value	0.120	0.050	<0.001	<0.001
ACE_ID * ACTN3 * Gender	effect size	<0.001	<0.001	<0.001	<0.001
	p-value	NaN	NaN	NaN	NaN
PTK2rs7843014	effect size	0.170	0.220	0.210	0.130
	p-value	<0.001	<0.001	<0.001	<0.001
PTK2rs7843014 * Gender	effect size	<0.001	<0.001	<0.001	<0.001
	p-value	NaN	NaN	NaN	NaN
PTK2rs7460	effect size	0.050	<0.001	<0.001	<0.001
	p-value	0.010	0.840	0.440	0.680
PTK2rs7460 * Gender	effect size	<0.001	<0.001	<0.001	<0.001
	p-value	NaN	NaN	NaN	NaN
PTK2rs7843014 * PTK2rs7460	effect size	0.040	0.010	0.080	0.050
	p-value	0.030	0.250	<0.001	0.010
PTK2rs7843014 * PTK2rs7460 * Gender	effect size	<0.001	<0.001	<0.001	<0.001
	p-value	NaN	NaN	NaN	NaN
TNCrs2104772	effect size	0.090	0.120	0.060	0.040
	p-value	<0.001	<0.001	0.010	0.070
TNCrs2104772 * Gender	effect size	0.060	0.010	0.060	0.070
	p-value	0.010	0.350	0.010	0.010

Table S2. *Genotype associations with aerobic fitness.* Summary of the effect sizes and p-values for the association of the five studied genotypes with parameters assessed during CPX-testing

main effects (>0.10)		*peak VO2*	*peak VO2_scaled*	*peak VCO2*	*peak VCO2_scaled*	*peak HF*	*peak P*	*peak*
P_scaled	*peak RER*	*peak VE*	*peak VT*	*peak AF*	*body height*	*body weight*	*age*	
ACE_ID	effect size	0.010	0.030	0.020	0.050	<0.001	0.020	0.030
	0.070	0.020	0.130	0.050	0.110	0.060	0.040	
	p-value	0.260	0.050	0.210	0.010	0.830	0.140	0.050
	<0.001	0.210	<0.001	<0.001	<0.001	<0.001	0.010	
ACE_ID * Gender	effect size	0.010	<0.001	0.010	<0.001	0.050	<0.001	<0.001
	0.010	<0.001	0.140	0.030	<0.001	<0.001	<0.001	
	p-value	0.300	0.310	0.240	0.330	<0.001	0.860	0.950
	0.110	0.480	<0.001	0.020	0.410	0.910	0.560	
ACTN3	effect size	0.010	0.010	0.010	0.010	0.020	0.020	0.020
	0.020	0.010	<0.001	<0.001	0.060	<0.001	0.090	
	p-value	0.480	0.440	0.230	0.380	0.090	0.080	0.170
	0.190	0.540	0.670	1.000	<0.001	0.770	<0.001	
ACTN3 * Gender	effect size	0.060	0.030	0.080	0.040	0.030	0.080	0.050
	0.020	0.050	0.010	0.030	0.010	<0.001	<0.001	
	p-value	<0.001	0.010	<0.001	<0.001	0.020	<0.001	<0.001
	0.030	<0.001	0.220	0.010	0.170	0.500	0.440	
ACE_ID * ACTN3	effect size	0.080	0.090	0.110	0.120	0.240	0.060	0.070
	0.030	0.090	0.060	0.110	0.140	0.020	0.110	
	p-value	<0.001	<0.001	<0.001	<0.001	<0.001	0.010	<0.001
	0.080	<0.001	<0.001	<0.001	<0.001	0.280	<0.001	
ACE_ID * ACTN3 * Gender	effect size	<0.001	<0.001	<0.001	<0.001	<0.001	<0.001	<0.001
	<0.001	<0.001	<0.001	<0.001	<0.001	<0.001	<0.001	
	p-value	NaN	NaN	NaN	NaN	NaN	NaN	NaN
	NaN	NaN	NaN	NaN	NaN	NaN	NaN	
PTK2rs7843014	effect size	0.090	0.110	0.150	0.160	0.020	0.040	0.030
	0.040	0.110	0.130	0.030	0.030	0.040	0.010	
	p-value	<0.001	<0.001	<0.001	<0.001	0.170	0.010	0.030
	0.010	<0.001	<0.001	0.050	0.050	0.010	0.290	
PTK2rs7843014 * Gender	effect size	0.010	0.080	0.010	0.080	<0.001	0.020	0.120
	<0.001	<0.001	0.020	<0.001	0.050	0.080	0.040	
	p-value	0.230	<0.001	0.130	<0.001	0.890	0.070	<0.001
	0.410	0.680	0.050	0.590	<0.001	<0.001	<0.001	
PTK2rs7460	effect size	0.050	0.020	0.060	0.030	0.020	0.050	0.020
	0.060	0.040	0.030	0.020	0.020	0.050	0.020	
	p-value	<0.001	0.130	<0.001	0.040	0.090	<0.001	0.190
	<0.001	0.010	0.040	0.190	0.110	0.010	0.140	
PTK2rs7460 * Gender	effect size	<0.001	0.030	<0.001	0.050	0.030	<0.001	0.050
	<0.001	0.010	0.080	<0.001	0.030	0.040	0.010	

	p-value	0.860	0.010	0.480	<0.001	0.020	0.610	<0.001
	0.870	0.110	<0.001	0.900	0.020	<0.001	0.100	
PTK2rs7843014 * PTK2rs7460	effect size	0.010	0.020	0.010	0.030	0.050	<0.001	0.040
	0.020	0.020	0.100	0.030	0.060	0.040	0.040	
	p-value	0.360	0.140	0.320	0.050	0.010	0.630	0.010
	0.080	0.170	<0.001	0.030	<0.001	0.010	0.020	
PTK2rs7843014 * PTK2rs7460 * Gender	effect size	<0.001	<0.001	<0.001	<0.001	<0.001	<0.001	<0.001
	<0.001	<0.001	<0.001	<0.001	<0.001	<0.001	<0.001	
	p-value	NaN	NaN	NaN	NaN	NaN	NaN	NaN
	NaN	NaN	NaN	NaN	NaN	NaN	NaN	
TNCrs2104772	effect size	0.060	0.260	0.080	0.250	<0.001	0.010	0.140
	<0.001	0.090	0.030	0.230	0.100	0.110	0.070	
	p-value	<0.001	<0.001	<0.001	<0.001	0.880	0.320	<0.001
	0.880	<0.001	0.020	<0.001	<0.001	<0.001	<0.001	
TNCrs2104772 * Gender	effect size	0.100	0.020	0.110	0.040	0.020	0.070	0.040
	0.050	0.070	0.060	0.060	0.100	0.180	0.050	
	p-value	<0.001	0.120	<0.001	0.010	0.180	<0.001	0.010
	<0.001	<0.001	<0.001	<0.001	<0.001	<0.001	<0.001	

Table S3. *Genotype associations with metabolic economy.* Summary of the effect sizes and p-values for the association of the five studied genotypes with the assessed slopes between cardiorespiratory parameters and power output as assessed during CPX-testing

main effects (>0.10)		VO2_slope	VCO2_slope	HF_slope	RER_slope	VE_slope	VT_slope	AF_slope
ACE_ID	effect size	<0.001	0.030	0.040	0.080	0.010	0.090	0.030
	p-value	0.690	0.070	0.010	<0.001	0.560	<0.001	0.050
ACE_ID * Gender	effect size	0.010	0.010	0.010	0.070	<0.001	0.170	<0.001
	p-value	0.170	0.210	0.270	<0.001	0.570	<0.001	0.470
ACTN3	effect size	0.020	0.010	<0.001	0.020	0.020	0.030	0.010
	p-value	0.090	0.470	0.730	0.080	0.080	0.050	0.530
ACTN3 * Gender	effect size	<0.001	<0.001	0.010	<0.001	0.010	<0.001	<0.001
	p-value	0.910	0.410	0.240	0.610	0.270	0.380	0.870
ACE_ID * ACTN3	effect size	0.020	0.050	0.030	0.010	0.050	0.020	0.080
	p-value	0.200	0.010	0.120	0.620	0.010	0.190	<0.001
ACE_ID * ACTN3 * Gender	effect size	<0.001	<0.001	<0.001	<0.001	<0.001	<0.001	<0.001
	p-value	NaN	NaN	NaN	NaN	NaN	NaN	NaN
PTK2rs7843014	effect size	0.060	0.140	0.050	0.050	0.030	0.020	0.010
	p-value	<0.001	<0.001	<0.001	0.010	0.050	0.200	0.330
PTK2rs7843014 * Gender	effect size	<0.001	<0.001	0.040	<0.001	0.020	0.020	<0.001
	p-value	0.430	0.340	<0.001	0.320	0.030	0.030	0.590
PTK2rs7460	effect size	<0.001	0.010	0.040	0.010	0.020	0.020	0.030
	p-value	0.850	0.400	0.020	0.220	0.190	0.200	0.040

PTK2rs7460 * Gender	effect size	0.010	<0.001	0.080	<0.001	0.040	0.050	<0.001
	p-value	0.290	0.420	<0.001	0.710	<0.001	<0.001	0.370
PTK2rs7843014 * PTK2rs7460	effect size	<0.001	<0.001	0.180	0.010	0.060	0.040	0.020
	p-value	0.680	0.670	<0.001	0.350	<0.001	0.010	0.180
PTK2rs7843014 * PTK2rs7460 * Gender	effect size	<0.001	<0.001	<0.001	<0.001	<0.001	<0.001	<0.001
	p-value	NaN	NaN	NaN	NaN	NaN	NaN	NaN
TNCCrs2104772	effect size	0.060	0.080	0.030	<0.001	0.060	0.020	0.120
	p-value	<0.001	<0.001	0.040	0.750	<0.001	0.150	<0.001
TNCCrs2104772 * Gender	effect size	0.020	0.010	<0.001	<0.001	0.010	0.030	0.010
	p-value	0.160	0.240	0.620	0.920	0.350	0.030	0.260
