# Supplementary figures and images for: Association of Gene Variants for Mechanical and Metabolic Muscle Quality with Cardiorespiratory and Muscular Variables Related to Performance in Skiing Athletes
Source: Genes (Basel). 2022 Oct 5;13(10):1798. doi: 10.3390/genes13101798 (PMC9602077; doi:10.3390/genes13101798)

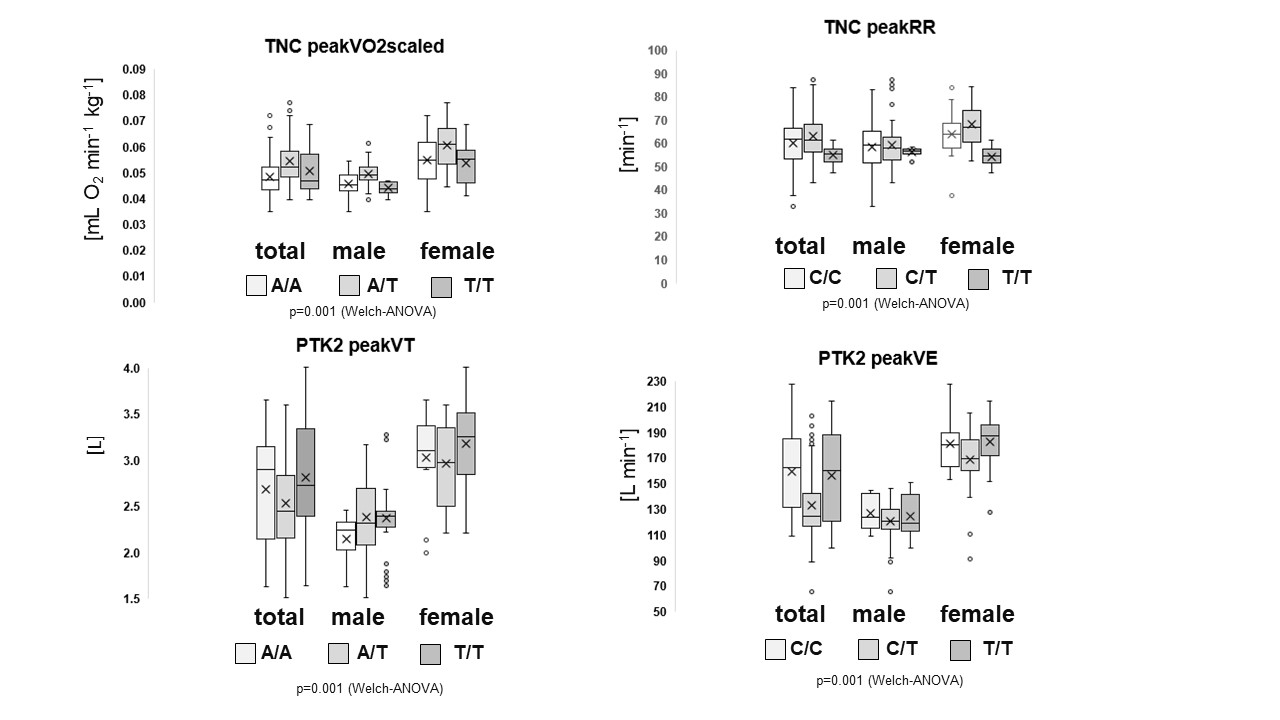

Supplement: Supplementary file 1 [file genes-13-01798-s001.zip › 1912388-Figure S1.jpg]

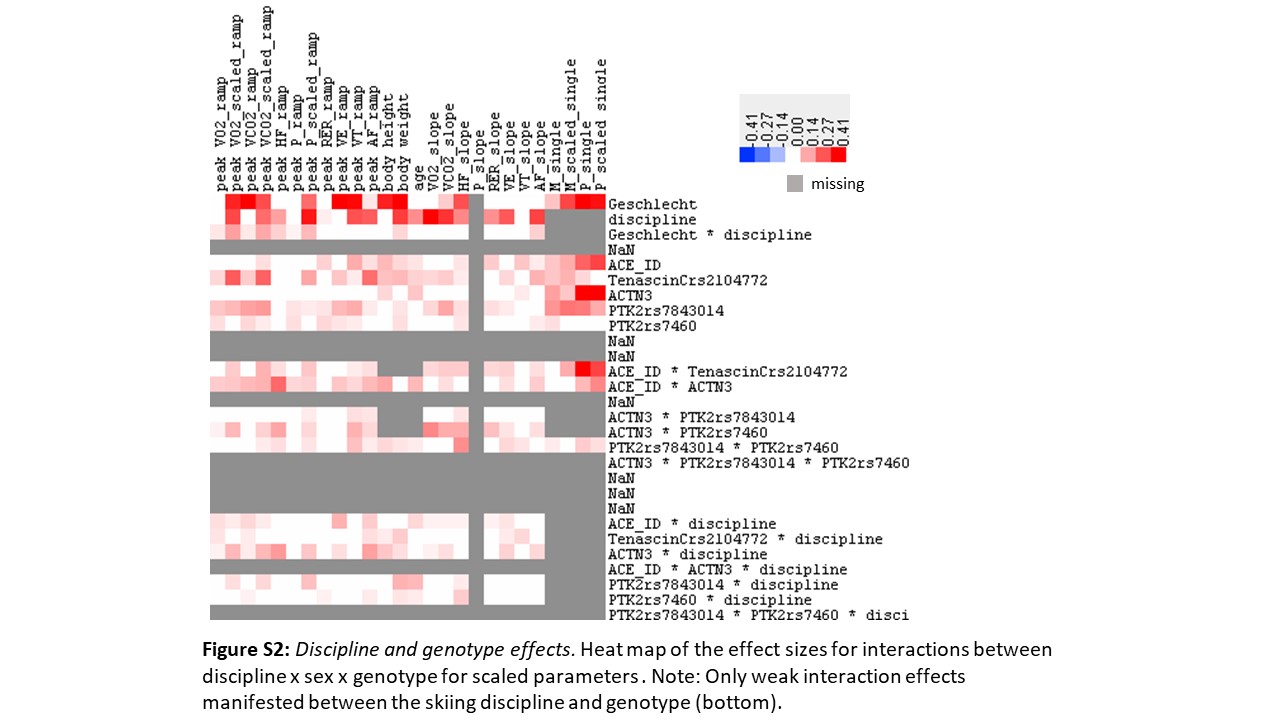

Supplement: Supplementary file 1 [file genes-13-01798-s001.zip › 1912388-Figure S2.jpg]
